# Functional Role of miR-138-5p and miR-200b-3p in Testicular Germ Cell Tumors: Molecular Insights into Seminoma and Teratoma Pathogenesis

**DOI:** 10.3390/ijms26168107

**Published:** 2025-08-21

**Authors:** Fatemeh Hooshiar, Hossein Azizi, Mahla Masoudi, Thomas Skutella

**Affiliations:** 1 Department of Stem Cells and Cancer, College of Biotechnology, Amol University of Special Modern Technologies, Amol 46158-63111, Iran; ftmehooshiar97@gmail.com (F.H.); mahlamasoudii@gmail.com (M.M.); 2 Institute for Anatomy and Cell Biology, Medical Faculty, University of Heidelberg, Im Neuenheimer Feld 307, 69120 Heidelberg, Germany; thomas.skutella@uni-heidelberg.de

**Keywords:** genomics, germ cell tumors, molecular pathways, miRNA profiling, seminoma, teratoma

## Abstract

This study aims to investigate the molecular mechanisms underlying germ cell tumors (GCTs), focusing specifically on seminomas and teratomas. By analyzing gene expression profiles and miRNA interactions, the goal is to identify key regulatory miRNAs and signaling pathways that differentiate these tumor types and could serve as important regulators for therapy development. Raw data for seminomas and teratomas were extracted from the GEO database, and gene hubs were identified using STRING and Gephi. Signaling pathways and functional annotations were analyzed using miRPathDB, while miRNA–gene interactions were explored via miRWalk. Hub miRNAs were filtered and confirmed using miRDB. This study highlights significant changes in gene expression diversity between tumor and normal gonadal tissues, providing insights into the molecular dynamics of seminomas and teratomas. Distinctions between seminomas and teratomas were identified, shifting the focus toward miRNAs to discover more precise and novel therapeutic approaches. The hub genes of seminomas and teratomas were identified separately. MiRNAs targeting these hub genes were also determined and confirmed. These miRNAs collectively influence essential oncogenic pathways—confirming hsa-miR-138-5p as a regulator of pathways such as Hippo signaling, transcriptional misregulation in cancer, and microRNA cancer signaling in seminomas, and hsa-miR-200b-3p as a regulator of p53 signaling, T cell receptor signaling, and pathways including PI3K/AKT, MAPK/ERK, and Wnt/β-catenin in teratomas—confirming their potential as promising candidates for subtype-specific therapeutic intervention. MiRNAs identified through bioinformatics analyses, and their predicted regulatory roles in key oncogenic pathways, represent potential therapeutic targets or regulators of biological processes. However, further experimental validation is needed to confirm these findings.

## 1. Introduction

Germ cell tumors (GCTs) are a diverse group of neoplasms originating from germ cells, affecting individuals across various ages and genders. They represent over 95% of testicular cancers and are primarily classified into two major types, seminomas and non-seminomas, each exhibiting distinct clinical behaviors, histological features, and treatment responses [1,2]. Seminomas account for approximately half of all testicular germ cell tumors and are characterized by slow growth and uniform cellular morphology. They predominantly affect young men aged 25 to 45 years [3]. In contrast, non-seminomas—which include subtypes such as teratomas, embryonal carcinomas, and others—are generally more aggressive and have a higher likelihood of early metastasis [4]. Teratomas are particularly complex tumors composed of tissues derived from all three embryonic germ layers (ectoderm, mesoderm, and endoderm), contributing to their unique biological and therapeutic challenges [5]. Recent epidemiological data indicate that the incidence of testicular GCTs has been steadily increasing worldwide, emphasizing the urgent need for improved diagnostic and therapeutic approaches. Despite advances in surgical techniques and chemotherapy regimens, treatment resistance and relapse remain significant clinical challenges, particularly for non-seminomatous tumors such as teratomas [6]. Advances in sequencing technologies and the rapid decline in associated costs have enabled comprehensive genomic profiling of hundreds of cancer-related genes. These tools allow researchers to identify gene expression patterns, key signaling pathways, and potential biomarkers that improve diagnostic accuracy and enable the development of personalized treatment strategies [7]. Among the molecular factors implicated in tumorigenesis, microRNAs (miRNAs) have emerged as powerful regulators of gene expression. These small, non-coding RNA molecules modulate critical cellular processes including proliferation, differentiation, apoptosis, and genomic stability. Dysregulation of miRNAs has been linked not only to cancer initiation but also to tumor progression, metastasis, and response to therapy. Therefore, miRNAs represent promising candidates both as biomarkers and therapeutic targets [8].

To date, however, the specific miRNA profiles and their functional roles in seminomas and teratomas remain incompletely understood. Identifying the distinct miRNA-mediated regulatory networks that govern these tumor types can provide new insights into their pathogenesis and uncover novel molecular targets for therapeutic intervention. Given the clinical and molecular importance of distinguishing seminomas from teratomas, this study aims to identify key miRNAs and associated signaling pathways that underpin the biology of these tumors. The findings are expected to provide deeper insights into their molecular mechanisms and highlight precise therapeutic targets for improved clinical management.

## 2. Results

**Figure 1:**
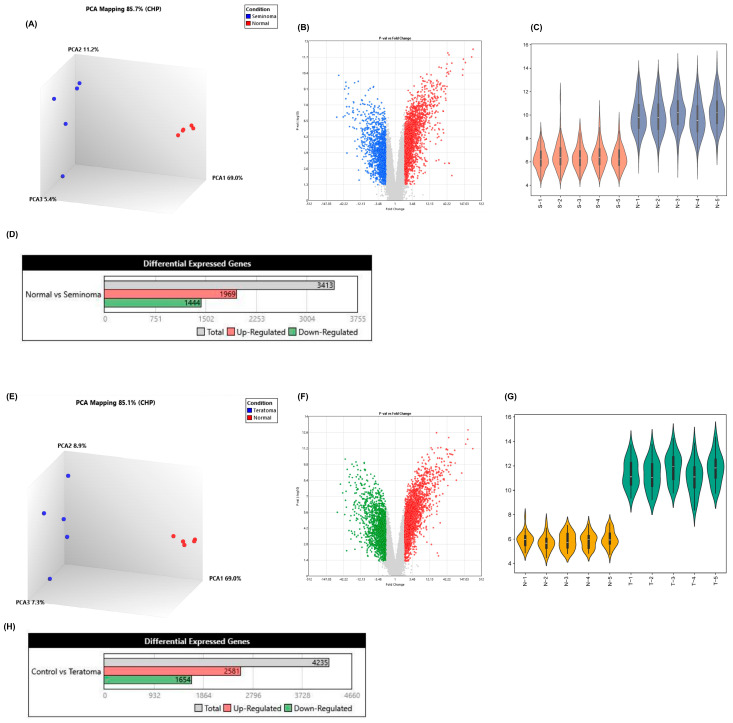
Transcriptomic analysis of seminoma and teratoma. (**A**) 3D PCA plot showing clear separation between seminoma (blue) and normal (red) samples (PC1 = 69.0%, PC2 = 11.2%, PC3 = 5.4%); (**B**) volcano plot of seminoma versus normal tissues (|log_2_FC| > 1, FDR < 0.05) (Blue dots: underexpressed genes; Red dots: overexpressed genes.); (**C**) violin plots of expression distributions: seminoma (S-1–S-5) versus normal (N-1–N-5); (**D**) bar chart of differentially expressed genes in seminoma; (**E**) 3D PCA plot for teratoma (blue) versus normal (red) samples (PC1 = 69.0%, PC2 = 8.9%, PC3 = 7.3%); (**F**) volcano plot of teratoma versus normal tissues (|log_2_FC| > 1, FDR < 0.05) (Green dots: underexpressed genes; Red dots: overexpressed genes.); (**G**) violin plots of expression distributions: normal (N-1–N-5) versus teratoma (T-1–T-5); (**H**) bar chart of differentially expressed genes in teratoma. These panels confirm distinct transcriptional profiles and extensive differential gene expression in both seminoma and teratoma compared to normal gonadal tissue.

After processing and visualization in the R environment, the comparison of the five seminoma and teratoma profiles individually with five control profiles representing normal gonadal tissues is shown. In PCA plots, seminoma and teratoma show clear distinctions from normal samples, indicating different expression profiles between these groups. However, the point distribution within each group suggests that seminomas may display a more uniform expression pattern than teratomas, as evidenced by the tighter clustering observed in the seminoma plots (Figure 1A,D). Volcano plot analysis revealed significant transcriptomic differences between seminoma and teratoma. Seminoma showed a higher number of upregulated genes (1969 out of 3413 DEGs) (Figure 1D and Supplementary File S1) with a more uniform expression pattern, suggesting activation of oncogenic pathways such as proliferation and immune regulation. In contrast, teratoma displayed a more balanced and heterogeneous distribution of 4235 DEGs (2581 upregulated, 1654 downregulated) (Figure 1H and Supplementary File S2), reflecting its complex tissue composition. These findings indicate that seminoma may respond better to targeted therapies, while teratoma may require broader therapeutic approaches. Violin plots were used to compare the distribution of gene expression levels between tumor and normal tissues in both teratoma and seminoma groups. In teratoma samples (Figure 1G), the tumor group (T1–T5, green) displayed markedly higher gene expression levels compared to normal gonadal tissues (N1–N5, orange). The tumor samples showed a broader range and a higher median expression, indicating strong transcriptional upregulation in teratoma. In contrast, seminoma samples (Figure 1C) showed a more moderate increase in gene expression (S1–S5, red) relative to the normal tissues (N1–N5, blue), with a narrower distribution range and slightly elevated median values. This pattern suggests that while both tumor types demonstrate increased gene activity compared to normal tissues, teratoma exhibits more pronounced transcriptional deregulation, likely reflecting its greater molecular complexity.

**Figure 2:**
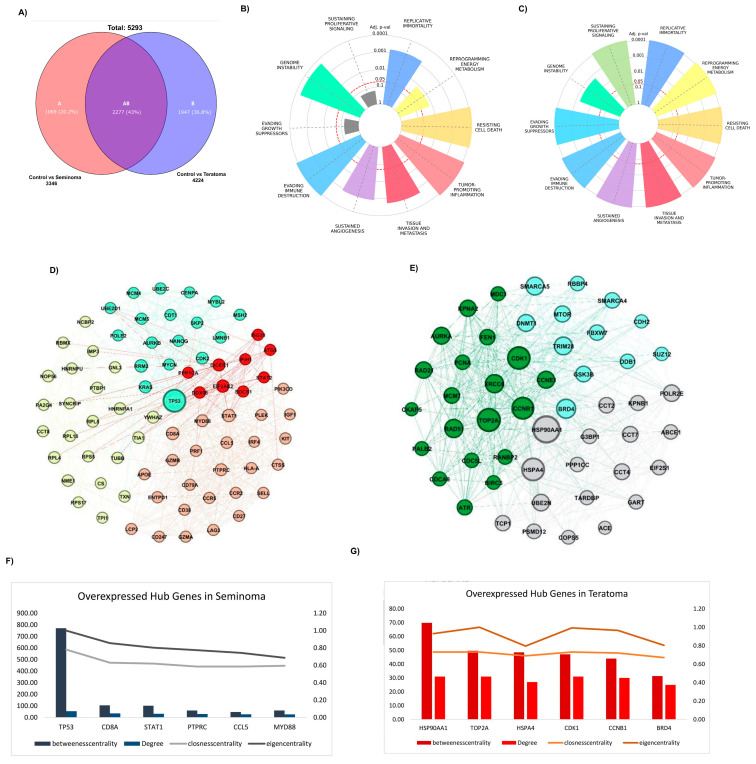
Hub gene identification in seminoma and teratoma. (**A**) Venn diagram of DEGs; hallmark enrichment for specific genes of seminoma (**B**) and teratoma (**C**) hubs, showing top cancer-related pathways (–log_10_ adj *p*-value); PPI networks for seminoma (**D**) and teratoma (**E**) hub genes; centrality metrics (betweenness, degree, closeness, eigenvector) for overexpressed hub genes in seminoma (**F**) and teratoma (**G**), highlighting top influencers.

To identify genes uniquely expressed in seminoma and teratoma as well as those common in both, a Venn diagram was constructed (Figure 2A). This analysis revealed 1069 genes specific to seminoma and 1947 genes unique to teratoma, with 2277 genes shared between the two tumor types. Given that this study focuses on tumor-specific genes, the expression patterns of the shared genes are provided separately in Supplemental Figure S1 and Supplementary File S3, and further analysis of these common genes was not pursued. The biological pathways associated with these tumor-specific genes were explored using Hallmark pathway enrichment analyses for each group. Figure 2B,C present the Hallmark pathway charts for seminoma and teratoma, respectively. In seminoma (Figure 2B), pathways related to genome instability, sustained proliferative signaling, evading growth suppressors, and reprogramming energy metabolism were prominently enriched, indicating active cell proliferation and genetic alterations characteristic of this tumor type. In contrast, teratoma (Figure 2C) showed stronger enrichment in pathways linked to tissue invasion and metastasis, tumor-promoting inflammation, and resistance to cell death, reflecting its aggressive phenotype and histological complexity. Protein–protein interaction (PPI) networks were constructed separately for the tumor-specific genes of seminoma and teratoma using the STRING database and visualized via Gephi software. The seminoma gene network was partitioned into four distinct clusters (Figure 2D), each representing functional modules with strong intra-cluster connections. The teratoma network, more complex, was divided into three clusters (Figure 2E). These networks illustrate the intricate functional relationships among the hub genes within each tumor type. Finally, the centrality and importance of hub genes within these networks were quantitatively assessed using multiple network metrics including degree, betweenness centrality, closeness centrality, and eigenvector centrality. Figure 2F,G show the comparative analyses of the top hub genes in seminoma and teratoma networks, respectively. Key genes such as *TP53*, *CD8A*, *STAT1*, *PTPRC*, *CCL5*, and *MYD88* dominated the seminoma network, whereas genes like *TRIM28*, *HSP90AA1*, *TOP2A*, *HSPA4*, *CDK1*, and *CCNB1* were more prominent in the teratoma network. These hub genes represent critical regulatory nodes and potential therapeutic targets unique to each tumor subtype.

**Figure 3:**
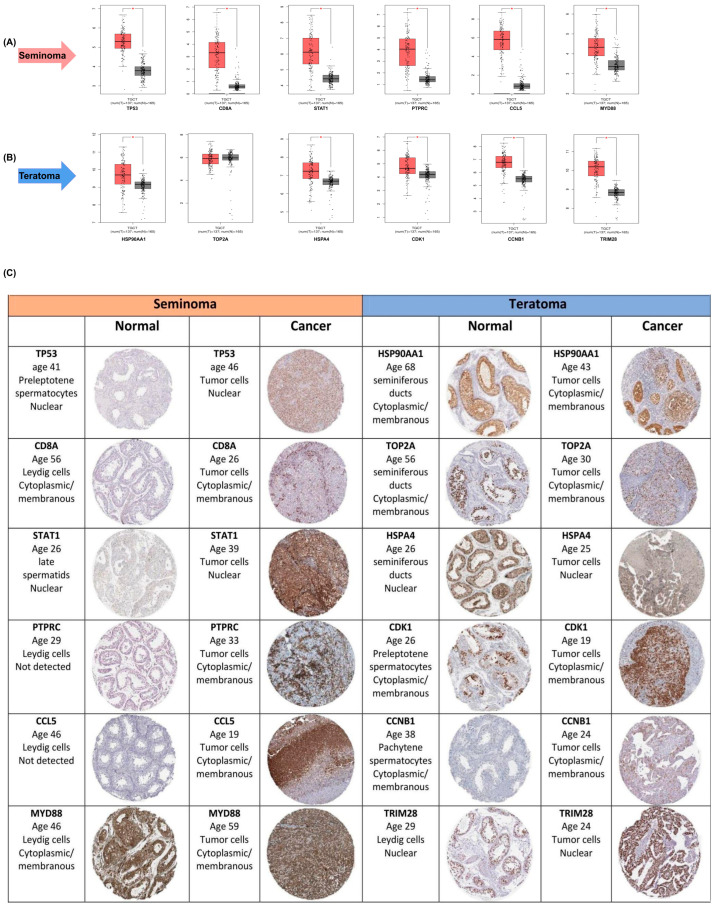
Validation of hub -gene expression in seminoma and teratoma. (**A**) Boxplots from GEPIA comparing mRNA levels of six seminoma hub genes—*TP53*, *CD8A*, *STAT1*, *PTPRC*, *CCL5*, and *MYD88*—in tumor versus normal testicular tissue (*p* < 0.01); (**B**) boxplots from GEPIA comparing mRNA levels of six teratoma hub genes—*HSP90AA1*, *TOP2A*, *HSPA4*, *CDK1*, *CCNB1*, and *TRIM28*—in teratoma versus normal tissue (*p* < 0.01); (**C**) representative immunohistochemistry images from the Human Protein Atlas showing protein expression for the same hub genes in normal and cancerous tissues. These data confirm both transcript- and protein-level overexpression of key hub genes in their respective tumor subtypes. Asterisks (*) indicate statistically significant differences (*p* < 0.05).

To corroborate these findings at the transcriptomic level, we employed the GEPIA database for expression validation. Boxplot analyses from GEPIA confirmed significantly higher mRNA expression of these hub genes in seminoma (Figure 3A) and teratoma (Figure 3B) tumor samples compared to normal tissues (*p* < 0.01). The expression levels of key hub genes were assessed in both seminoma and teratoma datasets. All hub genes, except for TOP2A, showed significant differences in expression between tumor and normal tissues. Notably, TOP2A did not reach statistical significance, indicating that its expression may not be as critical to the development or progression of these two tumor types compared to the other hub genes, such as CDK1, CCNB1, and HSP90AA1. Furthermore, to validate the elevated expression of hub genes identified in seminoma and teratoma, we utilized immunohistochemistry (IHC) images from the Human Protein Atlas database, which demonstrated markedly increased protein levels of these genes in tumor tissues compared to normal counterparts (Figure 3C). Specifically, seminoma samples showed strong immunoreactivity for *TP53*, *CD8A*, *STAT1*, *PTPRC*, *CCL5*, and *MYD88*, while teratoma tissues exhibited heightened expression of *HSP90AA1*, *TOP2A*, *HSP4A*, *CDK1*, *CCNB1*, and *TRIM28*. This robust concordance between protein-level immunostaining and mRNA expression profiles reinforces the biological relevance of these hub genes in germ cell tumor pathogenesis. Collectively, these multi-layered validations underscore the pivotal role of these hub genes as critical regulators.

**Figure 4:**
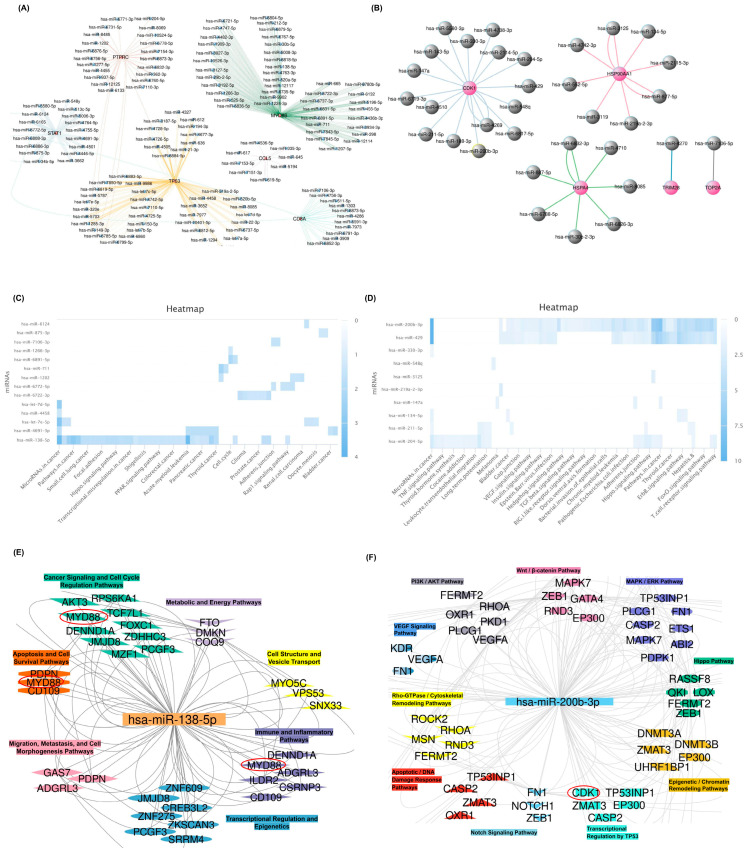
MiRNA–target gene networks and pathway enrichment in seminoma and teratoma. (**A**) The miRNA–gene interaction network for seminoma hub genes. (**B**) The miRNA–gene interaction network for teratoma hub genes highlights distinct modules centered. Each gray node indicates validated miRNA predicted to regulate a given seminoma hub gene. (**C**) Heatmap of seminoma-associated miRNA pathway enrichment. Rows represent individual miRNAs, columns show cancer-related pathways on the *x*-axis, and color intensity (blue gradient) corresponds to –log_10_ (adjusted *p*-value). (**D**) Heatmap of teratoma-associated miRNA pathway enrichment. Each row is a miRNA, each column is a pathway, and blue shades indicate higher enrichment. (**E**) Functional clustering of hsa-miR-138-5p targets in seminoma. (**F**) Functional clustering of hsa-miR-200b-3p targets in teratoma. Collectively, these panels demonstrate how hsa-miR-138-5p and hsa-miR-200b-3p arrange tumor-specific pathways by targeting distinct sets of hub genes.

The miRNA–gene interaction network for seminoma hub genes reveals a complex regulatory framework in which multiple key miRNAs target central oncogenes, including *MYD88*, *TP53*, *STAT1*, *CCL5*, and *CD8A* (Figure 4A). To ensure the accuracy of findings, only miRNAs that were experimentally validated in miRDB were included in the subsequent analysis. Similarly, the miRNA regulatory network associated with teratoma hub genes demonstrates distinct miRNA clusters targeting essential oncogenic regulators such as *CDK1*, *HSP90AA1*, *HSPA4*, *TRIM28*, and *TOP2A* (Figure 4B). These genes serve as critical nodes involved in diverse cancer-related pathways, underscoring their pivotal roles in seminoma and teratoma pathophysiology. To further explore the signaling pathways involving these miRNAs, the list of validated miRNAs was input into the mirPathDB database, and a heatmap of enriched signaling pathways was generated. This analysis highlighted the pathways in which these validated miRNAs are significantly involved, identifying miRNAs with the most prominent impact on cancer signaling. Pathway enrichment analysis visualized by heatmap for seminoma-associated miRNAs underscores significant involvement in multiple cancer-relevant signaling cascades. Notably, miRNAs, including hsa-miR-138-5p, showed strong enrichment in pathways such as microRNA cancer signaling, Hippo signaling, and transcriptional misregulation in cancer (Figure 4C). A direct interaction between *MYD88* and hsa-miR-138-5p has been experimentally validated in miRDB, confirming the regulatory role of this miRNA in seminoma. On the other hand, heatmap analysis of teratoma-associated miRNAs demonstrates pronounced enrichment in pathways implicated in cell cycle regulation, apoptosis, and DNA damage response. MiRNAs like hsa-miR-200b-3p exhibit strong pathway specificity, particularly affecting the p53 signaling pathway axis and T cell receptor signaling pathways (Figure 4D). A direct and experimentally validated interaction also exists between *CDK1* and hsa-miR-200b-3p in teratoma, further reinforcing the involvement of this miRNA in teratoma tumorigenesis. These findings highlight distinct molecular signatures between teratoma and seminoma.

The integrative functional clustering of seminoma-associated genes targeted by hsa-miR-138-5p highlights modular organization into key signaling pathways such as cancer signaling, apoptosis and cell survival, metabolic pathways, immune/inflammatory responses, and cytoskeletal dynamics (Figure 4E). Distinctly, central genes including *MYD88* are implicated in apoptosis and immune regulation, reinforcing the critical role of this miRNA in modulating tumor biology. For teratoma, the gene network targeted by hsa-miR-200b-3p reveals functional clusters associated with well-characterized oncogenic pathways including PI3K/AKT, MAPK/ERK, Wnt/β-catenin, and Hippo signaling (Figure 4F). Key genes such as *CDK1* occupy central positions within these clusters, emphasizing the widespread regulatory influence of hsa-miR-200b-3p in teratoma tumorigenesis.

**Figure 5:**
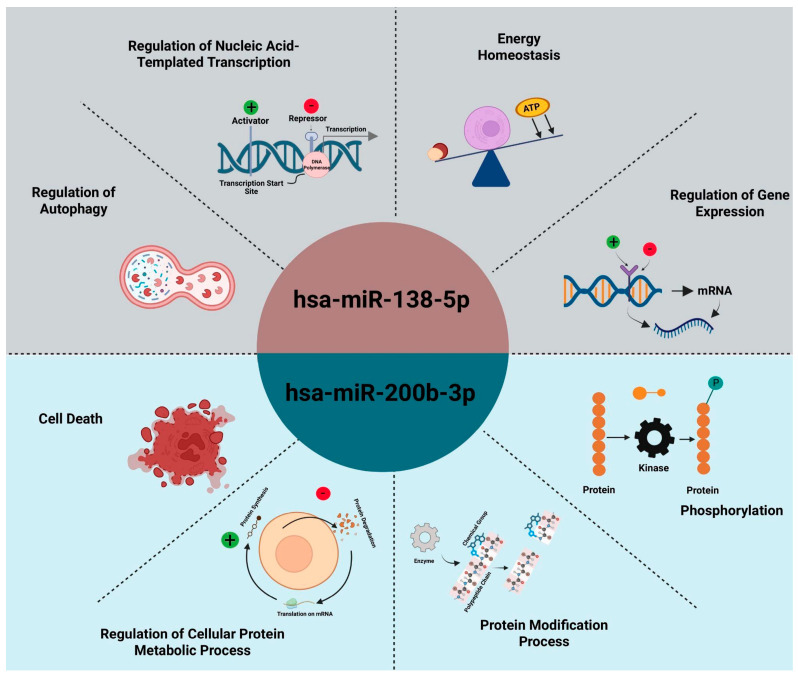
Overview of biological processes regulated by hsa-miR-138-5p and hsa-miR-200b-3p. Together, these functional domains illustrate how each miRNA orchestrates distinct but complementary aspects of tumor cell biology—hsa-miR-138-5p primarily governs transcriptional and metabolic homeostasis in seminoma, while hsa-miR-200b-3p directs kinase-driven signaling and protein turnover in teratoma.

Functional enrichment analysis revealed that the microRNAs hsa-miR-138-5p and hsa-miR-200b-3p regulate distinct sets of cancer-related biological processes. Hsa-miR-138-5p was found to influence transcriptional regulation, autophagy, energy homeostasis, and protein metabolism, including translation and phosphorylation, suggesting its role in modulating apoptosis, immune response, and cell cycle control—features that align with the immune-regulated, less aggressive nature of seminoma. In contrast, hsa-miR-200b-3p demonstrated strong associations with pathways involved in protein modification, kinase signaling, and regulation of cell death, which are central to oncogenic signaling networks such as MAPK/ERK and Wnt/β-catenin. These processes support the aggressive behavior and treatment resistance observed in teratomas. Overall, the divergent biological profiles of these miRNAs highlight their subtype-specific regulatory functions in testicular germ cell (Figure 5).

## 3. Discussion

This study provides a comprehensive comparative analysis of gene expression profiles, hub gene networks, and miRNA-mediated regulation in seminomas and teratomas—two major subtypes of testicular germ cell tumors (TGCTs). The results reveal profound molecular divergences between these tumor types, driven by distinct regulatory circuits that have direct implications for personalized therapeutic strategies. Transcriptomic analysis clearly demonstrated that both seminomas and teratomas exhibit significant transcriptional alterations compared to normal gonadal tissues. However, seminomas showed a more uniform gene expression pattern, characterized by 1969 upregulated out of 3413 differentially expressed genes (DEGs), suggesting a coherent oncogenic program that likely involves sustained proliferative signaling and immune evasion. This observation is supported by enriched Hallmark pathways related to genomic instability and cell cycle dysregulation—hallmarks commonly associated with seminomatous tumors [9]. In contrast, teratomas presented with 4235 DEGs, including 2581 upregulated genes, reflecting their heterogeneous tissue composition and complex histopathology. Pathway enrichment indicated stronger activation of metastasis-related cascades, tumor-promoting inflammation, and resistance to apoptosis, aligning with previous studies that describe adult teratomas as biologically aggressive and prone to malignant transformation [10].

A key highlight of this study is the identification of distinct hub gene networks in each tumor subtype. In seminoma, genes such as *TP53*, *STAT1*, *CD8A*, and *MYD88* emerged as central regulators. These genes are intricately linked with immune modulation and DNA damage response, reinforcing the immunogenic nature of seminoma. Conversely, the teratoma network was dominated by *CDK1*, *HSP90AA1*, *TRIM28*, and *TOP2A*—genes critically involved in mitotic progression, chromatin remodeling, and cellular stress response. Such divergence underscores the potential of subtype-specific gene targets for therapeutic intervention [11,12]. However, it is important to note that TOP2A did not show a statistically significant change in expression between tumor and normal tissues. This could indicate that TOP2A does not play as prominent a role in these specific tumor subtypes as initially hypothesized, or it may be regulated by alternative mechanisms not captured in this analysis. Further studies could investigate the precise role of TOP2A in these cancers, particularly focusing on its interaction with other molecular pathways.

Further strengthening the therapeutic potential of this molecular distinction, miRNA–gene interaction analysis revealed two key regulatory microRNAs: hsa-miR-138-5p in seminoma and hsa-miR-200b-3p in teratoma. Hsa-miR-138-5p was enriched in immune signaling, Hippo, and apoptosis pathways, suggesting it may act as a tumor suppressor by targeting genes such as *MYD88* and *STAT1*. Prior work has demonstrated the tumor-suppressive effect of miR-138-5p through regulation of *EZH2* and *CCND1* in other cancers [13].

In teratoma, hsa-miR-200b-3p displayed strong association with Wnt/β-catenin, MAPK/ERK, and T cell receptor signaling—pathways essential for cellular differentiation and immune escape. This miRNA has been shown to facilitate epithelial-to-mesenchymal transition (EMT) and metastasis by targeting *ZEB1* and *PTEN* [14], affirming its oncogenic role in pluripotent tumors such as teratomas. The strong concordance between mRNA and protein expression levels of hub genes, validated using the GEPIA and HPA databases, confirms the reliability of the computational findings and highlights actionable targets for therapy. Notably, overexpression of *HSP90AA1* in teratomas aligns with studies identifying *HSP90* inhibitors as potent agents in destabilizing oncogenic kinases and transcription factors [15]. Similarly, targeting TP53-regulating miRNAs in seminoma could enhance DNA damage responses and improve radiosensitivity, as suggested by recent immuno-genetic studies [16]. Although miR-138-5p and miR-200b-3p have been previously implicated in regulating oncogenes like *MYD88* and *CDK1* in various cancers, our study provides the first comprehensive analysis of these miRNAs in seminoma and teratoma. This research highlights their distinct roles in regulating tumor-specific signaling pathways, suggesting that miRNAs such as hsa-miR-138-5p and hsa-miR-200b-3p serve as potential therapeutic targets tailored to the unique molecular signatures of these tumor subtypes.

Taken together, the unique regulatory roles of hsa-miR-138-5p and hsa-miR-200b-3p closely reflect the distinct biological nature of seminoma and teratoma. These microRNAs do more than control essential cellular pathways—they capture the molecular signature of each tumor type. By connecting molecular mechanisms with pathological features, they offer promising avenues for deeper understanding and potential therapeutic development in germ cell tumors.

## 4. Materials and Methods

### 4.1. Data Extraction and Normalization

In this study, gene expression profiles were analyzed using the R programming environment with packages such as affy, gcrma, limma, and hgu133plus2.db. For preprocessing, normalization, and statistical analyses, the data were normalized using log base 2 transformation (log2). Initial quality assessment was performed through boxplots and histograms. Subsequently, data normalization was conducted using the RMA algorithm. Differential expression analysis was then carried out using the limma package with linear modeling and statistical tests adjusted for false discovery rate (FDR) to identify significant genes. The retrieved datasets underwent a comprehensive preparation process including quality control, removal of incomplete or defective samples, and normalization to ensure data integrity. The metadata of each dataset were carefully reviewed and cross-referenced with the original GEO study methods to ensure accuracy and reliability [17]. Two datasets, GSE18155 and GSE3218, were employed to analyze seminoma and teratoma samples, respectively.

### 4.2. Gene Expression Data Visualization and Analysis

For all differential expression gene (DEG) analyses, a threshold of adjusted *p*-value < 0.05 and absolute log2 fold change (|log2FC|) > 1 was set as the criterion for significant gene expression changes. These criteria were consistently applied across all analyses, figures, and tables to report only biologically meaningful results. To visualize gene expression patterns, heatmaps were generated using the ComplexHeatmap package in R, providing clear representations of gene expression across samples. Volcano plots illustrating significant genes based on fold change and statistical significance were created using the EnhancedVolcano package. Venn diagrams were employed to compare gene expression profiles of teratoma and seminoma samples, identifying unique and shared genes between these groups.

### 4.3. Network Construction and Validation

Protein–protein interaction (PPI) networks were identified and extracted using the STRING database (https://string-db.org/), accessed on 13 February 2025 [18]. The results of this analysis were imported into Cytoscape software (v3.10.1) to apply further filters based on gene parameters, including degree centrality, betweenness centrality, closeness centrality, and eigenvector centrality, in order to reduce the number of genes and visualize the PPI network) [19]. Gephi software (v0.9.2) was then used for gene clustering based on various gene parameters. By applying gene-related parameters within the network, gene influence was compared, and cancer-related hub genes were identified. Validation and expression analysis of target genes in cancerous and normal groups were performed using the GEPIA database (http://gepia.cancer-pku.cn/), which integrates data from TCGA (The Cancer Genome Atlas) and GTEx (Genotype-Tissue Expression) [20]. To investigate the protein expression of the hub genes identified in this study, immunohistochemistry (IHC) data and images from the Human Protein Atlas (HPA) database (https://www.proteinatlas.org) were utilized. This database provides comprehensive information on protein expression across various human tissues, including both cancerous and normal tissues [21].

### 4.4. MiRNA–Gene Interactions

Interactions between miRNAs and genes were initially identified and analyzed using the miRWalk database (http://mirwalk.umm.uni-heidelberg.de/, (accessed on 13 February 2025)) [22]. MiRNAs specifically interacting with hub genes (separately for seminoma and teratoma) were identified, and the frequency of these interactions was calculated within the R environment. Enriched signaling pathways associated with these miRNAs were analyzed using miRPathDB (https://mpd.bioinf.uni-sb.de/) [23]. To further confirm the identified interactions, the miRDB database (https://mirdb.org/) was used; only interactions confirmed by miRDB were retained for subsequent analyses, and unconfirmed interactions were excluded to enhance result accuracy [24].

### 4.5. Functional Clustering Analysis

Target miRNAs were initially submitted to the miRWALK database to retrieve all genes predicted to be regulated by these miRNAs. The resulting gene list was subsequently filtered based on validation status in the miRDB database to include only high-confidence targets for downstream analyses. These curated genes were then categorized according to their associated functional pathways, and miRNA–target interaction networks were constructed accordingly. Subsequently, biological processes associated with the identified miRNAs were elucidated using the miRPathDB database to further characterize their functional roles.

## 5. Conclusions

Through an integrated bioinformatics workflow combining miRWALK- and miRDB-validated miRNA–gene interactions with miRPathDB-based pathway enrichment and functional clustering, we have delineated distinct miRNA regulatory networks in seminoma and teratoma. In seminoma, hsa-miR-138-5p emerged as a master regulator by targeting hub gene such as *MYD88*, thereby modulating apoptosis, immune signaling, and cell-cycle pathways. In teratoma, hsa-miR-200b-3p prominently targets *CDK1* and other key oncogenes, exerting control over PI3K/AKT, MAPK/ERK, Wnt/β-catenin, and Hippo signaling. These findings not only clarify the pathway-specific roles of each miRNA but also highlight hsa-miR-138-5p and hsa-miR-200b-3p as promising candidates for subtype-specific therapeutic targeting. Future functional validation of these miRNA–mRNA interactions and preclinical testing of miRNA-based interventions may lead to more precise treatment strategies for testicular germ cell tumors.

## 6. Therapeutic Outlook and Study Limitations

The molecular distinction between seminomas and teratomas enables tailored therapeutic approaches: in seminomas, synthetic hsa-miR-138-5p mimics could reinforce apoptotic and anti-inflammatory pathways, while PD-1/PD-L1 checkpoint inhibitors may overcome *STAT1*- and *CD8A*-driven immune escape. In teratomas, combining *HSP90* inhibitors with standard chemotherapy and *CDK1* blockade (e.g., RO-3306) can arrest the cell cycle and induce apoptosis, and antagomiRs against hsa-miR-200b-3p offer a means to dampen proliferative and immune-evasive signaling. Multi-omics validation further supports these hub genes and miRNAs as both biomarkers and predictors of response. However, this study’s in silico reliance on miRDB, miRWalk, GEPIA, and STRING without experimental validation (qRT-PCR, Western blots, reporter assays), absence of clinical correlates (tumor stage, treatment outcomes), cross-sectional design, and omission of the tumor microenvironment (stromal, immune, vascular interactions) limit its translational impact. Addressing these gaps will require functional assays, incorporation of patient data, longitudinal sampling, and advanced single-cell or spatial transcriptomic analyses.

## Data Availability

The datasets analyzed during the current study are available from GEO (https://www.ncbi.nlm.nih.gov/geo/). 1. https://www.ncbi.nlm.nih.gov/geo/query/acc.cgi?acc=GSE18155. 2. https://www.ncbi.nlm.nih.gov/geo/query/acc.cgi?acc=GSE3218. And miRDB (https://mirdb.org/mirdb/index.html). 1. (hsa-miR-138-5p: *MYD88*) https://mirdb.org/cgi-bin/target_detail.cgi?targetID=2334121. 2. (hsa-miR-200b-3p: *CDK1*) https://mirdb.org/cgi-bin/target_detail.cgi?targetID=2394468, all accessed on 13 February 2025.

## Supplementary Materials

The following supporting information can be downloaded at: https://www.mdpi.com/article/10.3390/ijms26168107/s1.
