# Supplementary figures and images for: Functional Role of miR-138-5p and miR-200b-3p in Testicular Germ Cell Tumors: Molecular Insights into Seminoma and Teratoma Pathogenesis
Source: Int J Mol Sci. 2025 Aug 21;26(16):8107. doi: 10.3390/ijms26168107 (PMC12386904; doi:10.3390/ijms26168107)

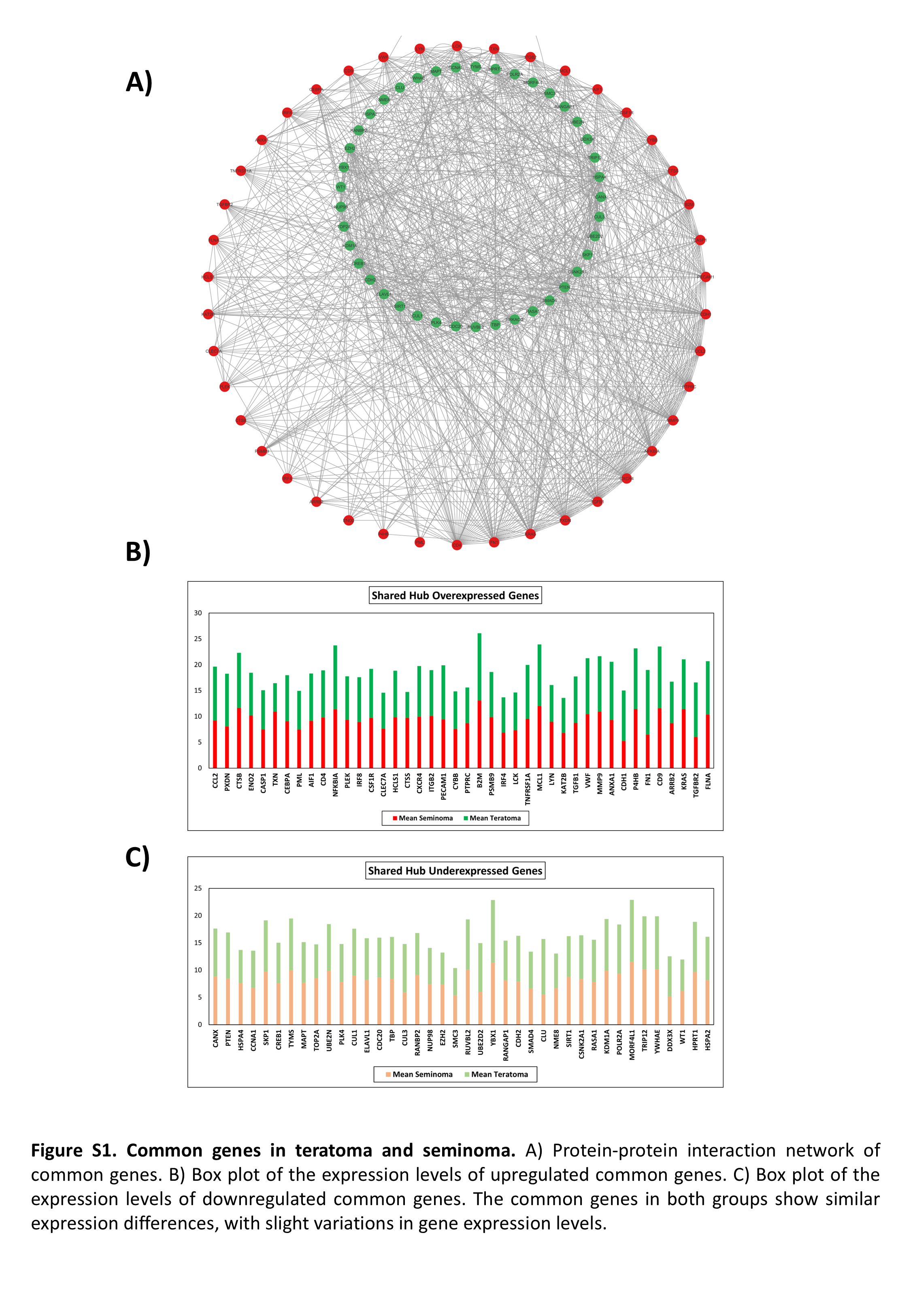

Supplement: Supplementary file 1 [file ijms-26-08107-s001.zip › Supplementary_Figure_S1.jpg]
